# Comment on “Association Between Dynapenic Obesity and Risk of Cardiovascular Disease: The Hisayama Study” by Setoyama et al. —The Authors' Reply

**DOI:** 10.1002/jcsm.13764

**Published:** 2025-03-05

**Authors:** Yu Setoyama, Takanori Honda, Toshiharu Ninomiya

**Affiliations:** ^1^ Department of Epidemiology and Public Health, Graduate School of Medical Sciences Kyushu University Fukuoka Japan; ^2^ Department of Orthopaedic Surgery, Graduate School of Medical Sciences Kyushu University Fukuoka Japan; ^3^ Center for Cohort Studies, Graduate School of Medical Sciences Kyushu University Fukuoka Japan

We would like to thank Drs. Wang and Huang for their helpful letter [1] regarding our paper [2].

As they pointed out, the generalizability of our findings is limited because this study was conducted in one town in Japan. Therefore, it would be desirable to verify the relationship between dynapenic obesity and cardiovascular disease (CVD) risk in various countries and regions with diverse backgrounds.

They also proposed additional subgroup analyses. We compared cardiovascular risk between dynapenic obesity and non‐dynapenic obesity by each covariate. As a result, the magnitude of the association of dynapenic obesity with the risk of CVD was stronger among participants with a smoking habit than in those without a smoking habit (*p* for heterogeneity = 0.04). Smoking has been reported to synergistically increase the risk of developing CVD when combined with hypercholesterolemia [3] and obesity [4]. In our study, the participants with dynapenic obesity had a higher frequency of hypercholesterolemia at baseline. These findings suggest that smoking may require more attention in individuals with dynapenic obesity, especially in middle‐aged individuals at high risk for CVD. In addition, we could not compare the risk in the subgroup with regular exercise habits because no CVD events were observed in the participants with dynapenic obesity and regular exercise habits. While this may have been due to chance given the small number of participants with dynapenic obesity who exercised regularly (*n* = 7), exercise has been shown to improve obesity and its complications [5, 6]. It may also prevent a worse prognosis in people with dynapenic obesity.

As also pointed out, we could not account for the changes in lifestyle factors such as diet, exercise, smoking and alcohol consumption during the follow‐up period in this study. It is certainly possible that cardiovascular risk decreased in participants with dynapenic obesity due to improvements in their lifestyle during the follow‐up period, whereas the opposite may have occurred in participants without dynapenic obesity. Consequently, the cardiovascular risk for those with dynapenic obesity at baseline against those without it may have been underestimated in this study. The impact of lifestyle changes on the association between dynapenic obesity and CVD needs to be verified in future studies.

Finally, we would like to thank Drs. Wang and Huang again for their valuable suggestions on our study. We hope that our study will contribute to promoting health during middle and old age.

## Ethics Statement

The authors certify that they comply with the ethical guidelines for authorship and publishing in the *Journal of Cachexia, Sarcopenia and Muscle* [7].

## Conflicts of Interest

The authors declare no conflicts of interest.
